# Adsorption Behavior of Toxic Carbon Dichalcogenides (CX_2_; X = O, S, or Se) on *β*_12_ Borophene and Pristine Graphene Sheets: A DFT Study

**DOI:** 10.3390/nano12193411

**Published:** 2022-09-29

**Authors:** Mahmoud A. A. Ibrahim, Amna H. M. Mahmoud, Gamal A. H. Mekhemer, Ahmed M. Shawky, Mahmoud E. S. Soliman, Nayra A. M. Moussa

**Affiliations:** 1 Computational Chemistry Laboratory, Chemistry Department, Faculty of Science, Minia University, Minia 61519, Egypt; 2 School of Health Sciences, University of KwaZulu-Natal, Westville, Durban 4000, South Africa; 3 Science and Technology Unit (STU), Umm Al-Qura University, Makkah 21955, Saudi Arabia; 4 Molecular Bio-Computation and Drug Design Research Laboratory, School of Health Sciences, University of KwaZulu-Natal, Westville, Durban 4000, South Africa

**Keywords:** graphene, borophene, carbon dichalcogenides, adsorption process, DFT

## Abstract

The adsorption of toxic carbon dichalcogenides (CX_2_; X = O, S, or Se) on *β*_12_ borophene (*β*_12_) and pristine graphene (GN) sheets was comparatively investigated. Vertical and parallel configurations of CX_2_⋯*β*_12_/GN complexes were studied herein via density functional theory (DFT) calculations. Energetic quantities confirmed that the adsorption process in the case of the parallel configuration was more desirable than that in the vertical analog and showed values up to −10.96 kcal/mol. The strength of the CX_2_⋯*β*_12_/GN complexes decreased in the order CSe_2_ > CS_2_ > CO_2_, indicating that *β*_12_ and GN sheets showed significant selectivity for the CSe_2_ molecule with superb potentiality for *β*_12_ sheets. Bader charge transfer analysis revealed that the CO_2_⋯*β*_12_/GN complexes in the parallel configuration had the maximum negative charge transfer values, up to −0.0304 *e*, outlining the electron-donating character of CO_2_. The CS_2_ and CSe_2_ molecules frequently exhibited dual behavior as electron donors in the vertical configuration and acceptors in the parallel one. Band structure results addressed some differences observed for the electronic structures of the pure *β*_12_ and GN sheets after the adsorption process, especially in the parallel configuration compared with the vertical one. According to the results of the density of states, new peaks were observed after adsorbing CX_2_ molecules on the studied 2D sheets. These results form a fundamental basis for future studies pertaining to applications of *β*_12_ and GN sheets for detecting toxic carbon dichalcogenides.

## 1. Introduction

Recently, the emission of greenhouse gases and toxic molecules into the environment has gathered unprecedented attention from the scholarly community. These molecules might cause severe heart and lung conditions and contribute to the greenhouse impact and the destruction of the ozone layer [1,2,3,4]. Among these harmful molecules, carbon dioxide (CO_2_) is a crucial gas because of its high concentration in the atmosphere as a result of the combustion of petroleum, coal, and other fossil fuels [5]. Carbon disulfide (CS_2_) is another toxic gas that adversely affects human health. It was documented that exposure to CS_2_ gas leads to many problems, including paucity of vitamin B_6_ and an increase in heart attack risk [6,7]. In the same vein, the carbon diselenide (CSe_2_) molecule is well recognized as a highly toxic molecule with unpleasant properties [8,9]. Because of its high toxicity, the CSe_2_ molecule must be handled with utmost care [10]. Many researchers have accordingly focused their efforts on developing various effective sensors for monitoring such toxic molecules.

Two-dimensional (2D) materials are a topic of interest for sensing purposes due to their vital physical and chemical properties. Graphene (GN), the first 2D form of carbon produced experimentally in 2004 [11], has received sustained attention due to its superior optical and mechanical properties [12,13,14,15,16,17]. Based on the electronic properties of 2D materials, the GN sheet was previously nominated as a Dirac material and could be recognized by defining its pseudomobility energy edges, energy spectrum surrounding the Fermi energy, and zero-energy confined state modes [18].

Crucially, GN has been used in many fields such as gas sensors [19], energy production [20], and spintronic devices [21]. The utilization of pristine and doped GN in detecting toxic gases, such as CO, NO, NO_2_, and NH_3_, has been investigated [14,22,23].

In addition to GN, many 2D materials have been developed, like molybdenum disulfide [24,25,26], phosphorene [27,28], silicene [29,30], and germanene [31,32], as active nanomaterials. Among the new 2D materials, borophene [33] has aroused the interest of the academic community. Indeed, borophene has been well characterized with versatile superior properties, including superconductivity, chemical complexity, low density, large bulk modulus, and high carrier mobility [34,35,36]. Borophene was earlier synthesized on a silver (111) surface in an ultrahigh vacuum [33]. Two phases of borophene have been observed using various deposition temperatures: the *β*_12_ phase with 1/6 vacancy concentration and the *χ*_3_ phase with 1/5 vacancy concentration [37]. From the literature, experimental and theoretical studies have revealed that all phases of borophene are metallic and exhibit superb electronic conductivity [37,38]. Further electronic properties of borophene as a Weyl 2D material, including high anisotropy and topological character, were also revealed [39,40].

The most stable type of borophene was reported to be *β*_12_ borophene (*β*_12_) [37,41]. Therefore, the sensing and trapping of greenhouse gases and other atmospheric pollutants (e.g., CO_x_, CH_4_, NH_3_, and NO_x_) using the *β*_12_ sheet has grown significantly [42,43,44,45].

**Figure 1:**
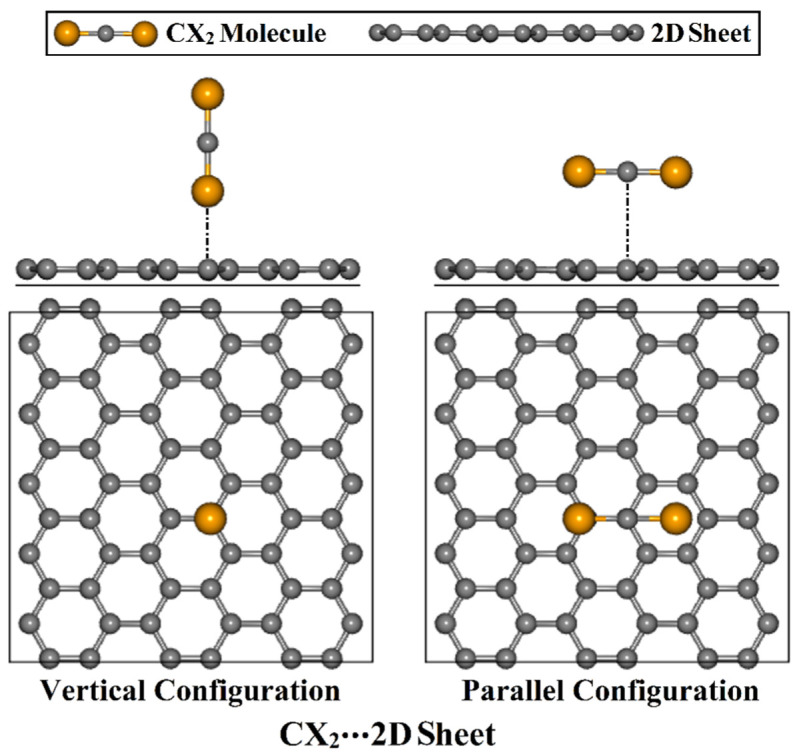
CSe_2_⋯GN complex as an illustration for the CX_2_⋯2D sheet complexes (where CX_2_ = CO_2_, CS_2_, or CSe_2_ and the 2D sheet = *β*_12_ or GN) within vertical and parallel configurations from the side and top representations.

Hence, this work was accordingly designed in order to deeply understand the potentiality of *β*_12_ and GN sheets to adsorb CX_2_ toxic molecules by employing density functional theory (DFT) calculations. In that spirit, the CX_2_⋯*β*_12_/GN complexes (CX_2_; X = O, S, or Se) were characterized in both vertical and parallel configurations (Figure 1). The geometric structures of CX_2_⋯*β*_12_/GN complexes were first subjected to relax calculations to obtain the minimum structures. The adsorption energies were then computed upon the relaxed structures of all the complexes under study. For most stable CX_2_⋯*β*_12_/GN complexes, charge transfer, electronic band structure, and density of state (DOS) analyses were performed to clearly elucidate the effect of the adsorption process on the features of the inspected 2D sheets. The findings of this study form a basis for future studies relevant to the applications of *β*_12_ and GN.

## 2. Computational Methods

Geometric optimization and energy calculations of the CX_2_⋯*β*_12_/GN complexes (CX_2_; X = O, S, or Se) were carried out in accordance with density functional theory (DFT) [46,47] via the Quantum ESPRESSO 6.4.1 package [48,49]. To describe the electronic interactions, the Perdew–Burke–Ernzerhof (PBE) exchange-correlation functional of the Generalized Gradient Approximation (GGA) was applied [50]. Ultrasoft pseudopotential was adopted for describing the interaction of valence electrons and the atomic cores [51]. The Grimme (DFT-D2) algorithm [52] was applied to correct the dispersion energy of the van der Waals interactions. In all the executed computations, the energy cutoff was set to 50 Ry, and the charge density cutoff was 500 Ry. The structures were optimized at energy and force convergence of 10^−5^ eV and 10^−4^ eV/Å, respectively. To sample and analyze the first Brillouin zone (BZ), Monkhorst–Pack grids were utilized with 6 × 6 × 1 *k*-points for geometric optimization and adsorption energy calculations. For the electronic structure calculations, 12 × 12 × 1 *k*-points were utilized. The Marzari–Vanderbilt smearing technique was performed to speed up the convergence [53]. To avoid interactions between neighboring atoms in the *z*-direction of the *β*_12_ and GN sheets, a vacuum layer with 20 Å was used. Supercells of 3 × 4 × 1 and 6 × 5 × 1 were modeled to calculate the adsorption energy of *β*_12_- and GN-containing complexes, respectively, containing 60 atoms in both sheets. For the CX_2_⋯*β*_12_/GN complexes, both vertical and parallel configurations were considered, as depicted in Figure 1. The adsorption energy (*E*_ads_) of all the studied complexes was computed using Equation (1):

(1) $$E_{\mathrm{ads}\;}=E_{{\mathrm{CX}}_{2}\cdots 2D\;\mathrm{sheet}}-\left(E_{{\mathrm{CX}}_{2}}+E_{2D\;\mathrm{sheet}}\right)$$

where $E_{{\mathrm{CX}}_{2}\cdots 2D\;\mathrm{sheet}}$, $E_{{\mathrm{CX}}_{2}}$, and $E_{2D\;\mathrm{sheet}}$ represent the energies of complexes, adsorbed CX_2_ molecules, and 2D sheets, respectively. The charge density difference (∆*ρ*) calculations were estimated using Equation (2):

(2) $$\Delta \rho =\rho_{\mathrm{total}}-\left(\rho_{{\mathrm{CX}}_{2}}+\rho_{2D\;\mathrm{sheet}}\right)$$

where $\rho_{\mathrm{total}}$, $\rho_{{\mathrm{CX}}_{2}}$, and $\rho_{2D\;\mathrm{sheet}}$ are the charge densities of complexes, adsorbed CX_2_ molecules, and 2D sheets, respectively. The Visualization for Electronic and Structural Analysis (VESTA) package was used to generate the ∆*ρ* maps [54]. Analysis of the Bader charge [55] was utilized to determine the charge transfer (*Q*_t_) to or from the 2D sheets according to Equation (3):

(3) $$Q_{t}=Q_{\mathrm{combined}\;2D\;\mathrm{sheet}}-Q_{\mathrm{isolated}\;2D\;\mathrm{sheet}}$$

where *Q*_combined 2D sheet_ indicates the total charge of the 2D sheets after the adsorption process, and *Q*_isolated 2D sheet_ represents the total charge of the 2D sheets before the adsorption process. To elucidate the electronic properties, the electronic band structure and the total and projected density of states (TDOS and PDOS) calculations were determined for the inspected 2D sheets. For the band structure calculations, high-symmetry points—namely, *Г* (0.0, 0.0, 0.0), *Y* (0.5, 0.0, 0.0), *S* (0.5, 0.5, 0.0), and *X* (0.0, 0.5, 0.0)—were selected, and 50 points were taken between each high-symmetry point.

## 3. Results and Discussion

### 3.1. Geometric Structures

**Figure 2:**
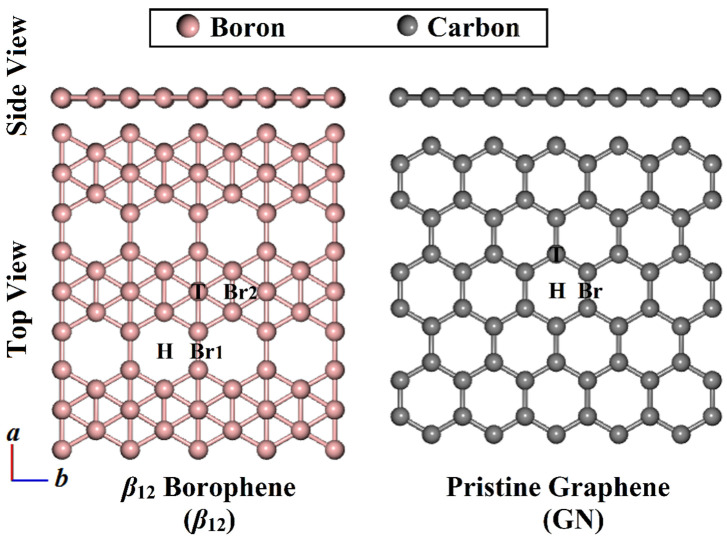
Side and top representations for optimized structures of 3 × 4 × 1 *β*_12_ and 6 × 5 × 1 GN with the modeled adsorption sites. T, Br, and H refer to top, bridge, and hollow sites.

The structures of the *β*_12_ and GN sheets were fully relaxed prior to the adsorption of the CX_2_ molecules on their surfaces, and the obtained structures are given in Figure 2.

After the relaxation of the *β*_12_ and GN sheets, the optimized lattice parameters of their primitive cells were *a* = 5.06 Å and *b* = 2.93 Å for *β*_12_ sheets (Figure 2), while *a* and *b* had a similar value of 2.47 Å in the case of GN sheets. The obtained findings were compatible with experimental and theoretical evidence [33,37,56,57].

According to the equilibrium structures displayed in Figure 2, four probable adsorption sites were identified in the *β*_12_ sheet—namely, top (T), hollow (H), and two bridge (Br1 and Br2) sites. For GN sheets, the top (T), bridge (Br), and hollow (H) sites were located above the carbon atom, C–C bond, and center of the hexagonal ring, respectively.

### 3.2. Adsorption Energy Calculations

**Figure 3:**
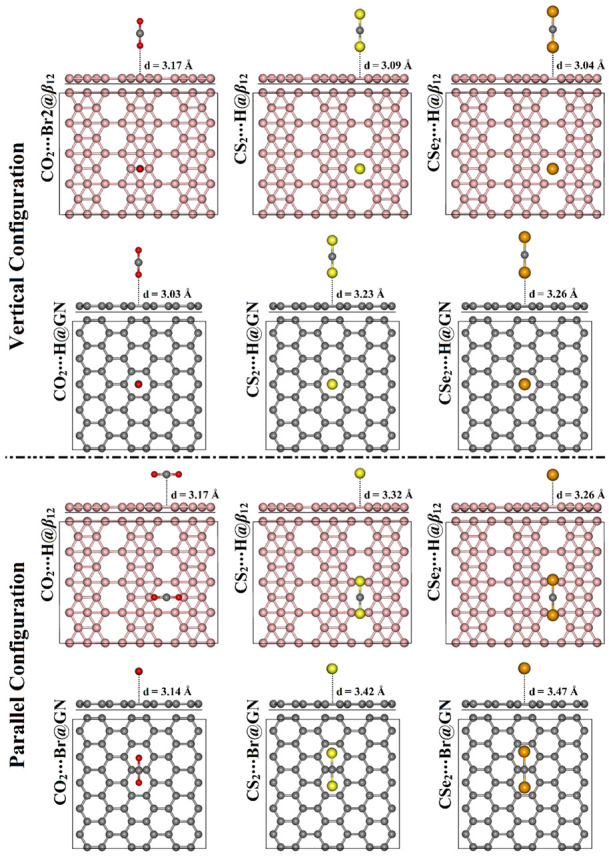
Side and top representations for the relaxed structures of the vertical and parallel configurations of the CX_2_⋯*β*_12_/GN complexes (where X = O, S, or Se) at the most favorable energetic sites. Equilibrium distances (d) are given in Å.

The adsorption process within the vertical and parallel configurations of the CX_2_⋯*β*_12_/GN complexes (where X = O, S, or Se) was unveiled at variant sites (Figure 2). The CX_2_⋯*β*_12_/GN complexes were first relaxed, and the obtained structures are given in Figure S1. The adsorption energies (*E*_ads_) for all relaxed complexes were then assessed and are gathered in Table 1. The most desirable relaxed structures of the CX_2_⋯*β*_12_/GN complexes are provided in Figure 3.

Apparently, the relaxed structures of the CX_2_⋯*β*_12_/GN complexes showed the ability of the *β*_12_ and GN sheets to adsorb toxic CX_2_ molecules (Figure S1), resulting in significant negative adsorption energies (Table 1). From Figure 3 and Table 1, the CX_2_ *β*_12_ and GN equilibrium distances were found to be in the ranges 3.04–3.49 and 3.03–3.55 Å, respectively.

For adsorption of the CX_2_ molecules on the *β*_12_ sheet in the vertical configuration, the CS_2_⋯ and CSe_2_⋯H@*β*_12_ complexes exhibited the most significant negative adsorption energies with values of −4.25 and −6.73 kcal/mol, respectively. For the CO_2_⋯*β*_12_ complexes, the most favorable adsorption energy value of −2.13 kcal/mol was obtained at the Br2 adsorption site of the *β*_12_ sheet (Table 1). In the parallel configuration, it was observed that the most favorable adsorption site on the *β*_12_ sheet for adsorbing all the CX_2_ molecules was the H@*β*_12_ site (Figure 3). Notably, the CSe_2_⋯H@*β*_12_ complex had the largest negative adsorption energy, followed by the CS_2_⋯H@*β*_12_ and CO_2_⋯H@*β*_12_ complexes, with values of −10.96, −6.53, and −4.42 kcal/mol, respectively (Table 1).

Similar to the CX_2_⋯*β*_12_ complexes, the most considerable negative adsorption energies were generally observed in the case of CSe_2_⋯GN complexes. In the case of the vertical configuration, the *E*_ads_ values of the studied complexes were noticed to decrease in the order CX_2_⋯H@GN > ⋯Br@GN > ⋯T@GN, showing the favorability of the H@GN site. Numerically, the *E*_ads_ values of CS_2_⋯H@GN, CS_2_⋯Br@GN, and CS_2_⋯T@GN were found to be –3.28, –3.14, and –3.13 kcal/mol, respectively. For the parallel configuration, the favorability of the CX_2_⋯GN complexes increased in the order of CX_2_⋯H@GN < CX_2_⋯T@GN < CX_2_⋯Br@GN. It was also observed that the CSe_2_⋯Br@GN complex with an equilibrium distance of 3.47 Å had the most significant *E*_ads_, with a value of −6.91 kcal/mol (Table 1).

Ultimately, all the studied carbon dichalcogenides (CX_2_; X = O, S, or Se) showed negative values of *E*_ads_, indicating greater preferentiality of the parallel configuration of the CX_2_⋯*β*_12_/GN complexes compared with the vertical one.

### 3.3. Band Structure Calculations

**Figure 4:**
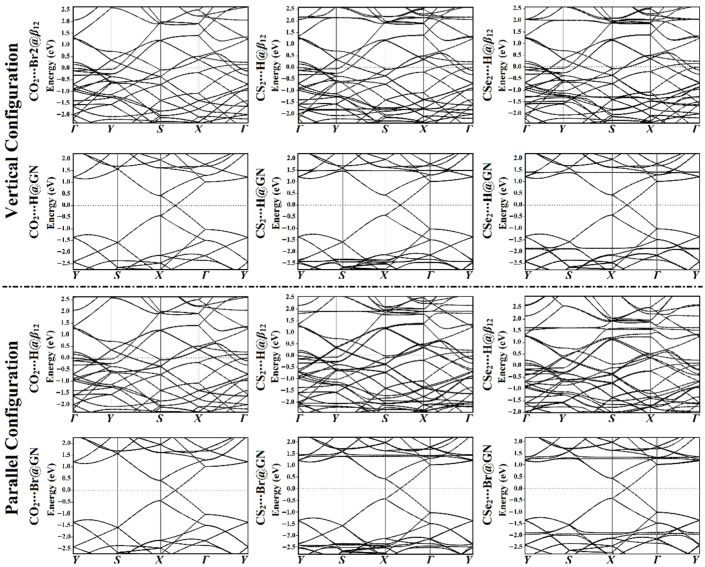
The plots of band structure for the relaxed structures of the vertical and parallel configurations of the CX_2_⋯*β*_12_/GN complexes (where X = O, S, or Se) at the most favorable energetic sites on the high-symmetry path of the Brillouin zone (BZ). The Fermi level is located at zero energy.

To trace the influence of the adsorbed CO_2_, CS_2_, and CSe_2_ molecules on the electronic features of the *β*_12_ and GN sheets, electronic band structure analysis was performed for the pure and combined 2D sheets. The PBE functional was used to calculate the band structure on the high-symmetry path of the BZ. The *Г-Y-S-X-Г* and *Y-S-X-Г-Y* paths were selected for band structures of the *β*_12_ and GN sheets, respectively. Figure S2 illustrates the band structures of the pure *β*_12_ and GN sheets. The electronic band structure calculations of the adsorbed CX_2_ molecules at the most favorable adsorption sites on the *β*_12_ and GN sheets were plotted and are shown in Figure 4.

According to Figure S2, the electronic band structure of the pure *β*_12_ surface showed a metallic character that was attributed to the presence of many bands that crossed the Fermi level along the high-symmetry path. In comparison, the Dirac point on the pure GN surface between points *X* and *Г* indicated that the GN exhibited a semiconductor amplitude that was consistent with that in previous work [17].

Figure 4 shows that the electronic properties of 2D sheets were marginally affected by the adsorption of the CO_2_ molecule in both studied configurations on the surfaces of the *β*_12_ and GN sheets (see Figure S2). Such an observation outlines that *β*_12_ and GN cannot be highly effective CO_2_ sensors, which agrees with evidence from previous studies [44,58]. In contrast, the adsorption of the CS_2_ and CSe_2_ molecules in vertical and parallel configurations on the *β*_12_ and GN sheets resulted in the appearance of many new bands in valence and conduction regions, as illustrated in Figure 4. The band structure plots showed that the bands of *β*_12_ moved far away from each other after adsorbing CS_2_ and CSe_2_ molecules, confirming the strong adsorption of these molecules on the *β*_12_ sheet. In detail, the resultant band structures after the adsorption of CS_2_ molecules on *β*_12_ showed extra conduction bands in the vertical and parallel configurations at around 2.3 and 1.8 eV, respectively. Besides this, new valence bands were noticed at –1.8 and –2.0 in the vertical and parallel configurations, respectively. For CS_2_⋯GN complexes, additional conduction and valence bands in both configurations were found at 1.5 and –2.5 eV, respectively. The band structures of the CSe_2_⋯*β*_12_ complexes strongly affirmed the more evident impact of the adsorbed CSe_2_ molecules on the electronic characteristics of the *β*_12_ sheet in the parallel configuration compared with the vertical analog. For adsorption of the CSe_2_ molecule in the parallel configuration at the H@*β*_12_ site, new bands were observed at 1.35, 1.55, and 1.65 eV in the conduction region. In addition, new bands at −0.4, −0.65, and −1.75 eV appeared in the valence region, as depicted in Figure 4.

Conspicuously, the results regarding the band structure demonstrated that the CSe_2_⋯*β*_12_ complexes were more favorable than the CS_2_⋯*β*_12_ complexes, as revealed by the bands that moved toward the Fermi level. To sum up, the electronic characteristics of the studied 2D sheets after adsorbing CX_2_ molecules were improved in the order CO_2_⋯*β*_12_/GN < CS_2_⋯*β*_12_/GN < CSe_2_⋯*β*_12_/GN, which is consistent with the results regarding the adsorption energies (Table 1). The appearance of new valence and conduction bands indicates the interaction of the CX_2_ molecules with the investigated 2D sheets. In addition, the presence of the Dirac point was not affected by the adsorption of CX_2_ molecules on the GN sheet, confirming the physical adsorption of CX_2_ on the GN sheet.

### 3.4. Charge Transfer Calculations

Bader charge analysis is considered an informative method for deducing the charge transfer throughout the adsorption process [55,59]. Thus, the charge transfer (*Q*_t_) was calculated for the vertical and parallel configurations of the CX_2_⋯*β*_12_/GN complexes at variant sites, and the results are given in Table 1. Negative and positive signs of the *Q*_t_ values indicate the transference of charge from the CX_2_ molecules to the 2D sheet and from the 2D sheet to the adsorbed CX_2_ molecules, respectively.

According to the *Q*_t_ values recorded in Table 1, adsorption of the CX_2_ molecules in the vertical configuration at different adsorption sites on the *β*_12_ sheet was noticed with negative values of *Q*_t_. For instance, the CO_2_⋯, CS_2_⋯, and CSe_2_⋯*β*_12_ complexes at the T site exhibited *Q*_t_ values of –0.0117, –0.0219, and –0.0127 *e*, respectively. These negative values demonstrate that the *β*_12_ sheet behaved as an electron acceptor.

The adsorption of the CO_2_ molecule on the *β*_12_ sheet, in the case of the parallel configuration, had negative values of *Q*_t_, demonstrating the electron donor character of the CO_2_ molecule. Moreover, the adsorption of the CS_2_ and CSe_2_ molecules on the *β*_12_ sheet showed positive *Q*_t_ values, proposing that the CS_2_ and CSe_2_ molecules acted as electron acceptors. Numerically, *Q*_t_ values of 0.0044 and 0.0513 *e* were shifted from the *β*_12_ sheet toward the CS_2_ and CSe_2_ molecules at the T@*β*_12_ site, respectively. In line with the adsorption energy results, the CSe_2_⋯H@*β*_12_ complex, the most favorable adsorption system in the parallel configuration, was found to have the largest positive *Q*_t_ value of 0.0724 *e*.

For the vertical configuration of the CX_2_⋯GN complexes, the H@GN site showed the highest negative *Q*_t_ values, consistent with its energetic preferentiality. Notably, all the CX_2_⋯GN complexes showed negative values of *Q*_t_, illustrating the potent ability of the GN sheet to accept electrons from the CX_2_ molecules.

Negative *Q*_t_ values were observed for the parallel configuration of the CO_2_⋯T/H/Br@GN complexes, while both negative and positive *Q*_t_ values were found at the studied adsorption sites in the case of the CS_2_⋯GN complexes. For adsorption of the CSe_2_ molecule, it can be seen that the charge was transferred from the GN sheet to the adsorbed CSe_2_, as indicated by the positive *Q*_t_ values shown in Table 1.

**Figure 5:**
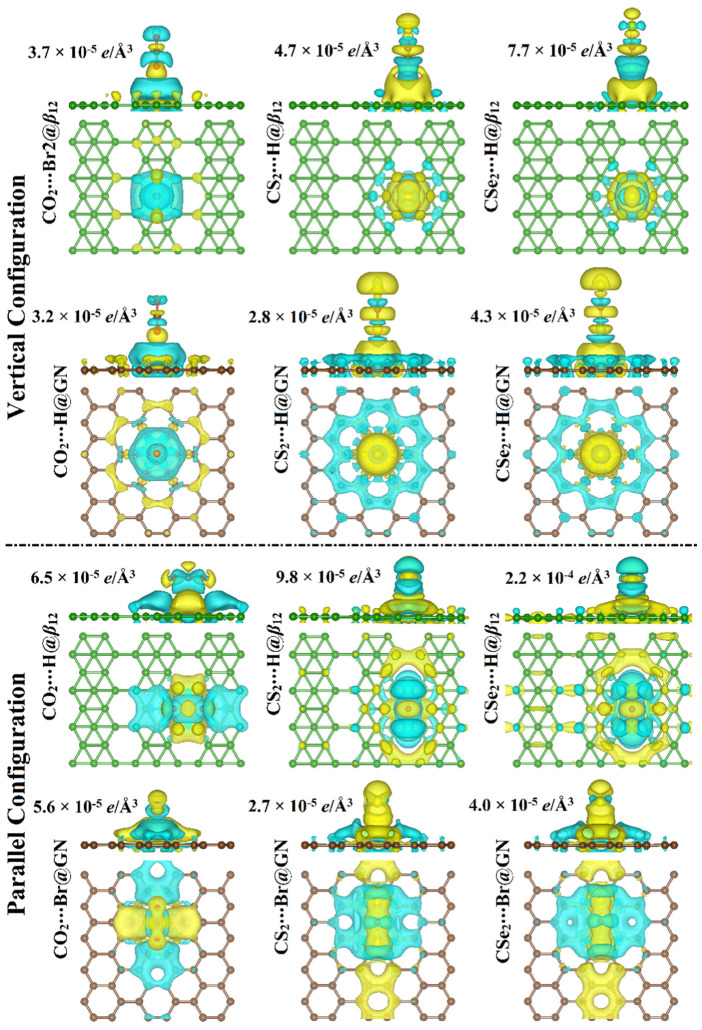
Side and top representations of the charge density difference (∆*ρ*) maps for the relaxed structures of the vertical and parallel configurations of the CX_2_⋯*β*_12_/GN complexes (where X = O, S, or Se) at the most favorable energetic sites. The charge accumulation and depletion are represented by the cyan and yellow colors, respectively. The isosurface values were determined in (*e*/Å^3^) for each complex. Dark green, brown, red, yellow, and pale green balls refer to boron, carbon, oxygen, sulfur, and selenium atoms, respectively.

At the most favorable energetic sites, the charge density difference (∆*ρ*) maps of the CX_2_⋯*β*_12_/GN complexes were plotted and are depicted in Figure 5.

As seen in Figure 5, the ∆*ρ* maps of the CX_2_⋯*β*_12_ complexes in the vertical configuration showed that the CX_2_ molecules acted as electron donors, as demonstrated by the negative *Q*_t_ values (Table 1). In the case of the parallel configuration, the charges were shifted from the CO_2_ to the *β*_12_ sheet, as seen from the charge accumulation region existing below the CO_2_ (cyan region) and corroborated by the negative *Q*_t_ value in Table 1 (−0.0271 *e*). A depletion region (i.e., yellow color) was clearly observed above the *β*_12_ sheet in the case of the adsorption of CS_2_ and CSe_2_ molecules in the parallel configuration, which was consistent with the positive *Q*_t_ values stated in Table 1. This observation emphasized that CS_2_ and CSe_2_ molecules have the potential to draw charge from the *β*_12_ sheet.

For the vertical configuration of the CX_2_⋯GN complexes, the existence of regions with accumulated charge ensures the adsorption of CX_2_ molecules on the *β*_12_ and GN sheets. The accumulated charge above the GN sheet within the CO_2_⋯ and CS_2_⋯GN complexes in the parallel configuration indicated that electrons were transferred from the CO_2_ and CS_2_ molecules to the GN sheet (Figure 5). In comparison, the depletion region above the GN sheet in the case of adsorption of the CSe_2_ molecule revealed the ability of the molecule to attract charge from the sheet.

To recapitulate, the Bader charge findings revealed that the charge was transferred from the CX_2_ molecules to the studied 2D sheets in the vertical configuration, indicating the electron-donating character of the CX_2_ molecules. The CO_2_⋯*β*_12_/GN complexes in the case of parallel configuration had the largest negative *Q*_t_ values, followed by CS_2_⋯*β*_12_/GN, then CSe_2_⋯*β*_12_/GN complexes. The small Q_t_ values confirmed physical adsorption between the CX_2_ molecules and the investigated 2D sheets. Consistent with the literature, the electronic properties of the *β*_12_ and GN sheets were changed by transferring electronic charge to or from the adsorbed molecules, indicating their potential for use as sensors [60,61,62].

### 3.5. Density of State Calculations

For pure and combined 2D sheets, density of state (DOS) analysis was carried out in terms of total and projected DOS (TDOS and PDOS). The TDOS and PDOS of the pure *β*_12_ and GN surfaces are illustrated in Figure S3.

From the data in Figure S3, the TDOS peaks of the pure *β*_12_ sheet at the Fermi level had high DOS, demonstrating that the *β*_12_ sheet had a metallic property. In the case of the GN surface, the TDOS peaks reached zero at the Fermi level, showing the presence of the Dirac point on the pure GN surface. The DOS results confirmed the band structures in Figure S2. For *β*_12_ and GN surfaces before adsorbing CX_2_ molecules, the major contributions to the DOS were ascribed to the PDOS of B*_p_* and C*_p_*, respectively.

**Figure 6:**
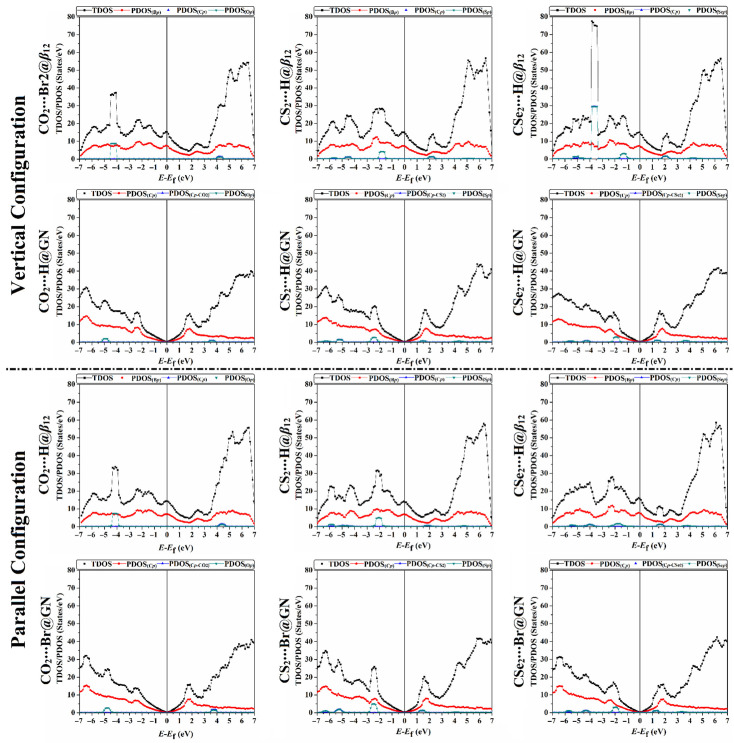
Total and projected densities of states (TDOS/PDOS) for the relaxed structures of the vertical and parallel configurations of the CX_2_⋯*β*_12_/GN complexes (where X = O, S, or Se) at the most favorable energetic sites. The contributions of the *p*-orbital for boron (B), carbon (C), oxygen (O), sulfur (S), and selenium (Se) atoms in the adsorption process are represented by B*_p_*, C*_p_*, O*_p_*, S*_p_*, and Se*_p_*, respectively.

Figure 6 shows the TDOS and PDOS for the *β*_12_ and GN sheets within the vertical and parallel configurations of the CX_2_⋯*β*_12_/GN complexes (where X = O, S, or Se) at the most favorable energetic sites. For adsorption of the CO_2_ molecule on the *β*_12_ sheet in both configurations, a significant hybridization between the PDOS of O*_p_* and B*_p_* peaks was observed in the valence region between −3.9 and −4.5 eV. In addition, the TDOS and PDOS peaks of adsorption of the CO_2_ molecule on the GN sheet in both configurations demonstrated the occurrence of a weak physical adsorption process. Hence, the appearance of the PDOS of O*_p_* peaks within the CO_2_⋯GN complexes in both configurations was noticed in the valence and conduction regions ranging from –4.5 to –5.6 eV and from 3.5 to 4.0 eV, respectively.

Conspicuously, the PDOS of S*_p_* was the major contributor to the adsorption within both modeled configurations of the CS_2_⋯*β*_12_/GN complexes. For both configurations of CS_2_⋯*β*_12_ and ⋯GN complexes, the appearance of the PDOS of S*_p_* peaks was observed in the valence region with values from −1.5 to −2.5 eV and from −2.0 to −3.0 eV, respectively.

For both configurations of CSe_2_⋯*β*_12_/GN complexes, the PDOS of Se*_p_* peaks appeared in the valence region in the range from −1.0 to −2.2 eV within the CSe_2_⋯*β*_12_ complexes and from −1.5 to −2.3 eV within the CSe_2_⋯GN complexes.

Overall, the DOS results outlined that the electronic properties of the *β*_12_ and GN sheets were changed after adsorbing the CX_2_ molecules in vertical and parallel configurations. The appearance of the new DOS peaks indicated the occurrence of adsorption of the CX_2_ molecules on the investigated 2D sheets.

## 4. Conclusions

The adsorption of toxic carbon dichalcogenides (CX_2_; X = O, S, or Se) on *β*_12_ and GN sheets was assessed via DFT calculations. After geometric relaxation, adsorption energy calculations and electronic analyses were carried out for vertical and parallel configurations of all CX_2_⋯*β*_12_/GN complexes. The favorability of CX_2_⋯*β*_12_/GN complexes was more obvious in the parallel configuration compared with the vertical one. The CSe_2_⋯H@*β*_12_ complex in the parallel configuration was the most promising complex, with an adsorption energy value of –10.96 kcal/mol. The electronic properties of the *β*_12_ and GN surfaces were notably changed after the adsorption of CS_2_ and CSe_2_ molecules. In comparison, the electronic characteristics of the *β*_12_ and GN surfaces were slightly changed after adsorbing the CO_2_ molecule. Based on Bader charge analysis, an electron-donating character was observed for all the CX_2_ molecules in vertical configuration within the CX_2_⋯*β*_12_/GN complexes. In comparison, the CS_2_ and CSe_2_ molecules acted as electron acceptors within the parallel configuration of the CS_2_⋯ and CSe_2_⋯*β*_12_/GN complexes. The results of the electronic band structure, TDOS, and PDOS demonstrated that adsorption of the CX_2_ molecules on the *β*_12_ and GN sheets boosted their electronic properties. The appearance of new bands and DOS peaks affirmed the interaction of the CX_2_ molecules with the investigated 2D sheets. Based on the findings of the present study, it appears promising to use the *β*_12_ and GN sheets as a suitable sensor for CX_2_ molecules, particularly CS_2_ and CSe_2_ molecules.

## Figures and Tables

**Table 1:** Adsorption energies (*E*_ads_, kcal/mol) and equilibrium distances (d, Å) for the vertical and parallel configurations of the CX_2_⋯*β*_12_/GN complexes (where X = O, S, or Se), in addition to the charge transfer differences (*Q*_t_, *e*) for the investigated 2D sheets after adsorbing the CX_2_ molecules.

2D Sheet	Adsorption Site ^a^	Carbon Dichalcogenides (CX_2_)								
		CO_2_			CS_2_			CSe_2_		
		*E*_ads_ (kcal/mol)	d (Å)	*Q*_t_ ^b^ (*e*)	*E*_ads_ (kcal/mol)	d (Å)	*Q*_t_ ^b^ (*e*)	*E*_ads_ (kcal/mol)	d (Å)	*Q*_t_ ^b^ (*e*)
**Vertical Configuration ^c^**										
***β*** **_12_**	T	−2.05	3.24	−0.0117	−3.54	3.35	−0.0219	−5.35	3.30	−0.0127
	H	−1.76	3.14	−0.0122	−4.25	3.09	−0.0199	−6.73	3.04	−0.0036
	Br1	--- ^d^	--- ^d^	--- ^d^	--- ^d^	--- ^d^	--- ^d^	--- ^d^	--- ^d^	--- ^d^
	Br2	−2.13	3.17	−0.0120	−3.47	3.34	−0.0233	−5.04	3.33	−0.0189
**GN**	T	−1.77	3.16	−0.0055	−3.13	3.31	−0.0077	−4.39	3.31	−0.0051
	H	−1.95	3.03	−0.0059	−3.28	3.23	−0.0097	−4.49	3.26	−0.0072
	Br	−1.79	3.14	−0.0055	−3.14	3.29	−0.0072	−4.39	3.30	−0.0040
**Parallel Configuration ^c^**										
***β*** **_12_**	T	−2.86	3.41	−0.0213	−5.49	3.49	0.0044	−8.54	3.43	0.0513
	H	−4.42	3.17	−0.0271	−6.53	3.32	0.0139	−10.96	3.26	0.0724
	Br1	−3.78	3.22	−0.0304	−6.29	3.38	−0.0039	−9.54	3.34	0.0335
	Br2	−2.96	3.37	−0.0225	−5.69	3.44	0.0044	−8.74	3.39	0.0484
**GN**	T	−3.64	3.19	−0.0155	−5.17	3.46	−0.0010	−6.81	3.49	0.0063
	H	−3.29	3.26	−0.0114	−4.83	3.52	0.0008	−6.45	3.55	0.0118
	Br	−3.77	3.14	−0.0146	−5.31	3.42	−0.0019	−6.91	3.47	0.0068

^a^ All adsorption sites on the *β*_12_ and GN surfaces are shown in Figure 2. ^b^ *Q*_t_ was estimated using Equation (3). ^c^ Figure S1 displays all relaxed structures of the CX_2_⋯*β*_12_/GN complexes. ^d^ No favorable adsorption was observed.

## Data Availability

Not applicable.

## Supplementary Materials

The following supporting information can be downloaded at: https://www.mdpi.com/article/10.3390/nano12193411/s1, Figure S1: Side and top representations for the relaxed structures of the vertical and parallel configurations of the CX_2_⋯*β*_12_/GN complexes (where X = O, S, or Se) at all the adsorption sites. Equilibrium distances (d) are given in Å. Figure S2: Band structure plots of *β*_12_ and GN sheets along the high-symmetry points of the Brillouin zone. The Fermi energy is located at zero-energy. Figure S3: Total and projected densities of states (TDOS/PDOS) plot for the pure surfaces of *β*_12_ and GN sheets, assuming the Fermi level as the reference level. The contributions of the *p*-orbital for boron (B) and carbon (C) atoms are represented by B*_p_* and C*_p_*, respectively.
