# Supplementary material for: Adsorption Behavior of Toxic Carbon Dichalcogenides (CX2; X = O, S, or Se) on β12 Borophene and Pristine Graphene Sheets: A DFT Study
Source: Nanomaterials (Basel). 2022 Sep 29;12(19):3411. doi: 10.3390/nano12193411 (PMC9565509; doi:10.3390/nano12193411)
Supplement: Supplementary file 1 [file nanomaterials-12-03411-s001.zip › nanomaterials-1934235-supplementary.pdf]

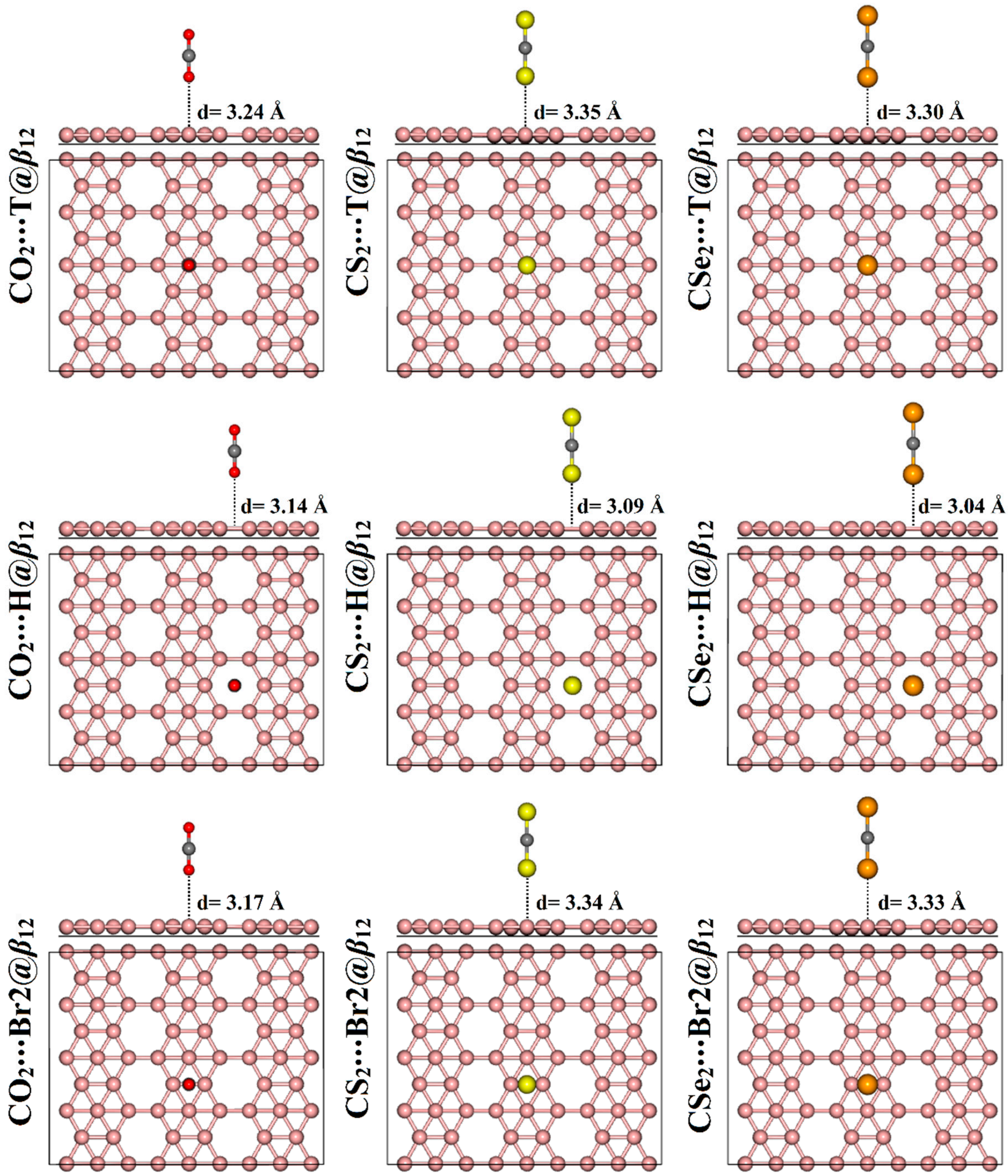

Figure S1. Side and top representations for the relaxed structures of the vertical and parallel configurations of the $CX_2 \cdots \beta_{12}$/GN complexes (where X = O, S, or Se) at all the adsorption sites. Equilibrium distances (d) are given in Å.

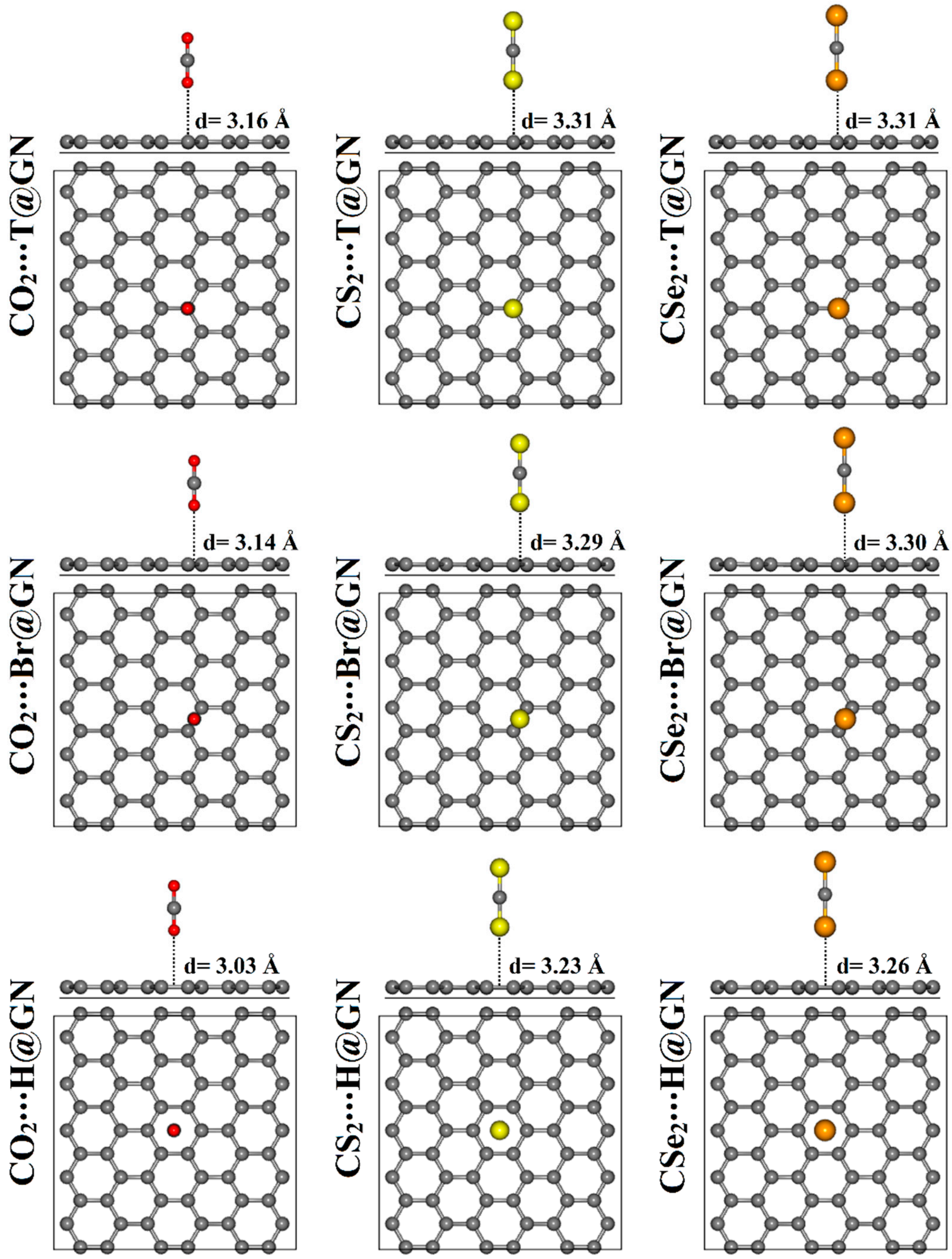

Figure S1. Continued.

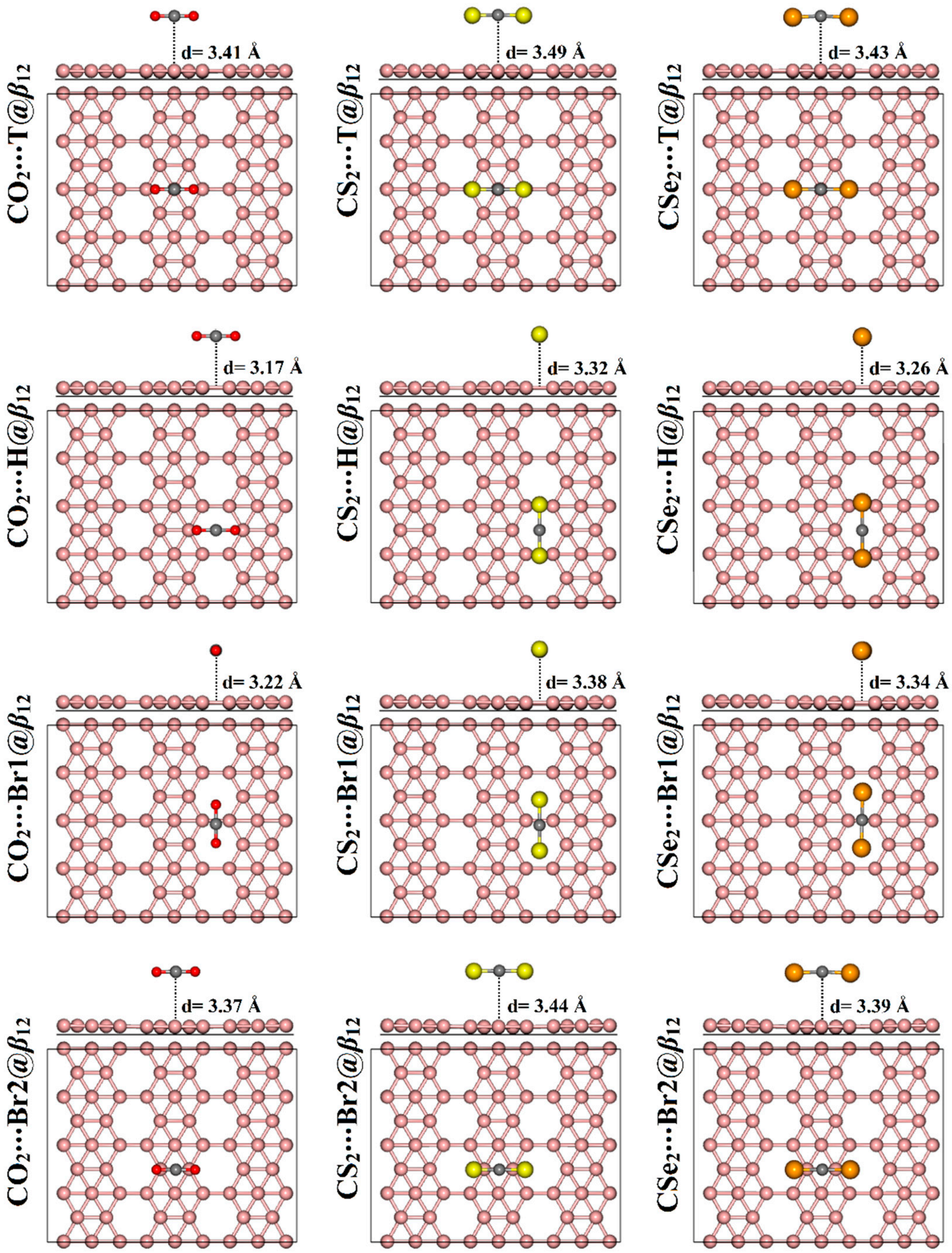

Figure S1. Continued.

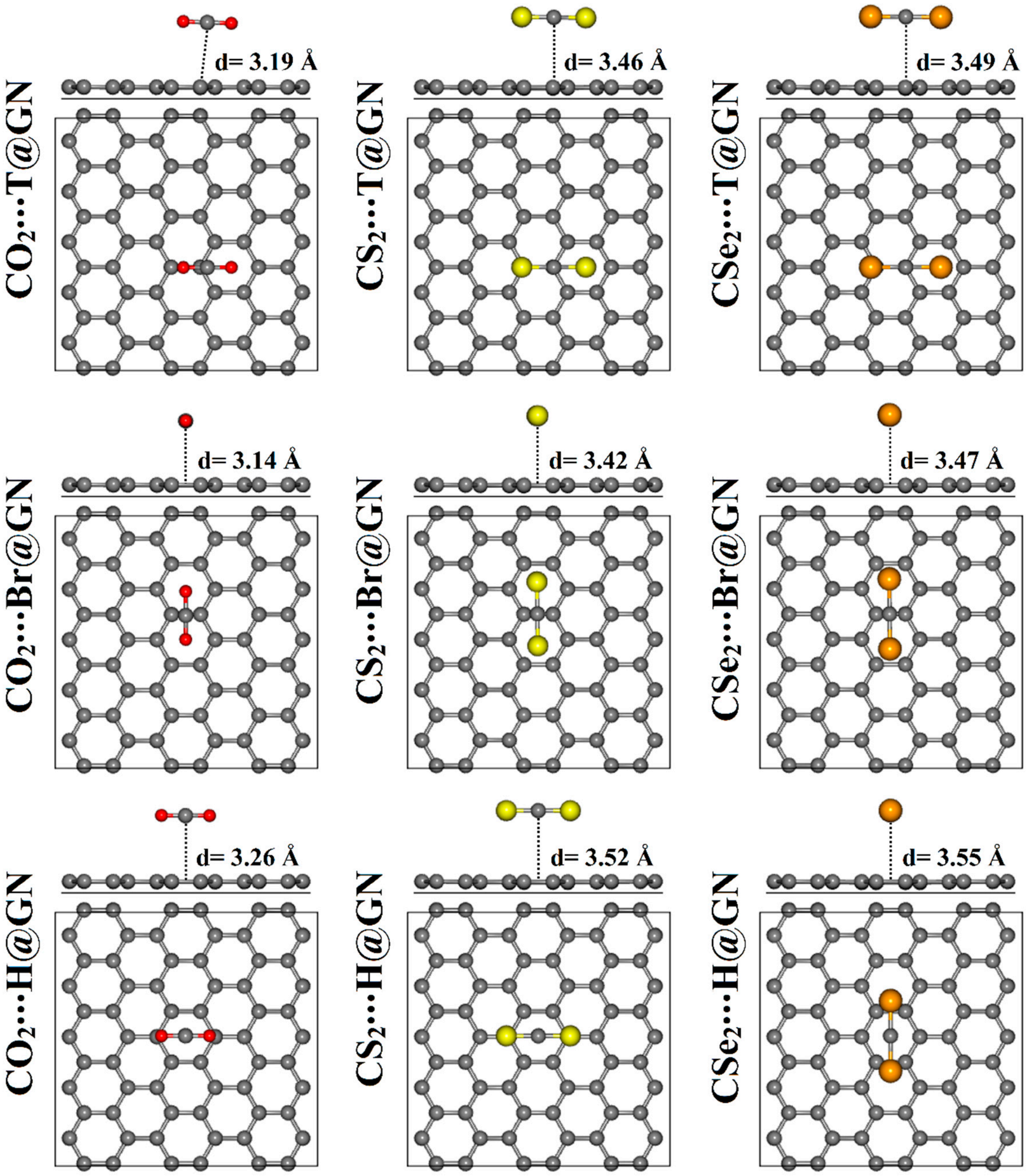

Figure S1. Continued.

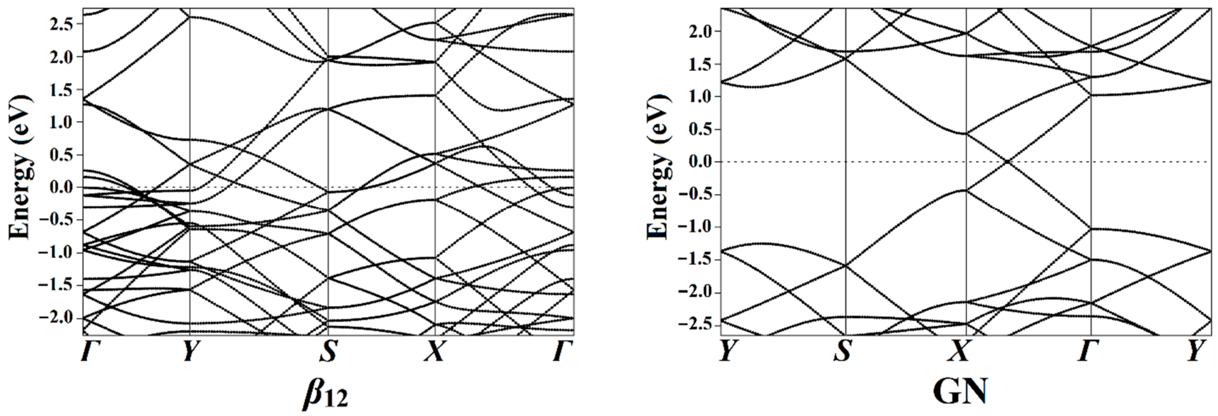

Figure S2. Band structure plots of β_{12} and GN sheets along the high-symmetry points of the Brillouin zone. The Fermi energy is located at zero-energy.

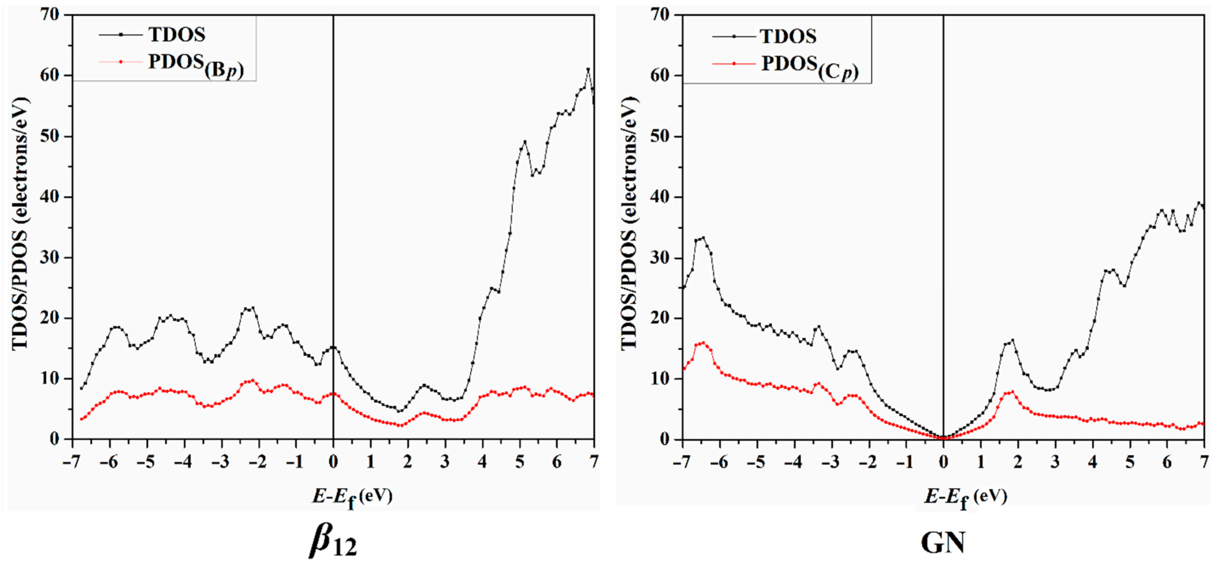

Figure S3. Total and projected densities of states (TDOS/PDOS) plot for the pure surfaces of β_{12} and GN sheets, assuming the Fermi level as the reference level. The contributions of the *p*-orbital for boron (B) and carbon (C) atoms are represented by B_p and C_p, respectively.

The crystallographic data of the relaxed β_{12} and GN sheets in the form of cif files.

For the relaxed β_{12} sheet:

```
#======================================================================
# CRYSTAL DATA
#----------------------------------------------------------------------
data_VESTA_phase_1

_chemical_name_common                  'XCrySDen XSF file'
_cell_length_a                         15.199867
_cell_length_b                         11.710008
_cell_length_c                         19.995638
_cell_angle_alpha                      90.000000
_cell_angle_beta                       90.000000
_cell_angle_gamma                      90.000061
_cell_volume                           3559.034827
_space_group_name_H-M_alt              'P 1'
_space_group_IT_number                 1

loop_
_space_group_symop_operation_xyz
   'x, y, z'

loop_
   _atom_site_label
   _atom_site_occupancy
   _atom_site_fract_x
   _atom_site_fract_y
   _atom_site_fract_z
   _atom_site_adp_type
   _atom_site_B_iso_or_equiv
   _atom_site_type_symbol
   B1         1.0     0.233555     0.127989     0.024911     Biso  1.000000 B
   B2         1.0     0.117864     0.127989     0.024911     Biso  1.000000 B
   B3         1.0     0.288221     0.252984     0.024911     Biso  1.000000 B
   B4         1.0     0.175698     0.252984     0.024910     Biso  1.000000 B
   B5         1.0     0.063185     0.252985     0.024911     Biso  1.000000 B
   B6         1.0     0.233555     0.377989     0.024911     Biso  1.000000 B
   B7         1.0     0.117864     0.377990     0.024911     Biso  1.000000 B
   B8         1.0     0.288221     0.502984     0.024911     Biso  1.000000 B
   B9         1.0     0.175698     0.502984     0.024910     Biso  1.000000 B
   B10        1.0     0.063185     0.502985     0.024911     Biso  1.000000 B
   B11        1.0     0.233555     0.627989     0.024911     Biso  1.000000 B
   B12        1.0     0.117864     0.627990     0.024911     Biso  1.000000 B
   B13        1.0     0.288221     0.752984     0.024911     Biso  1.000000 B
   B14        1.0     0.175698     0.752984     0.024910     Biso  1.000000 B
   B15        1.0     0.063185     0.752985     0.024911     Biso  1.000000 B
   B16        1.0     0.233555     0.877989     0.024911     Biso  1.000000 B
   B17        1.0     0.117864     0.877990     0.024911     Biso  1.000000 B
```

B18	1.0	0.288221	0.002984	0.024911	Biso	1.000000	B
B19	1.0	0.175698	0.002983	0.024910	Biso	1.000000	B
B20	1.0	0.063186	0.002985	0.024911	Biso	1.000000	B
B21	1.0	0.566889	0.127989	0.024911	Biso	1.000000	B
B22	1.0	0.451197	0.127990	0.024911	Biso	1.000000	B
B23	1.0	0.621554	0.252984	0.024911	Biso	1.000000	B
B24	1.0	0.509031	0.252984	0.024910	Biso	1.000000	B
B25	1.0	0.396519	0.252985	0.024911	Biso	1.000000	B
B26	1.0	0.566889	0.377989	0.024911	Biso	1.000000	B
B27	1.0	0.451197	0.377990	0.024911	Biso	1.000000	B
B28	1.0	0.621554	0.502984	0.024911	Biso	1.000000	B
B29	1.0	0.509031	0.502984	0.024910	Biso	1.000000	B
B30	1.0	0.396519	0.502985	0.024911	Biso	1.000000	B
B31	1.0	0.566888	0.627989	0.024911	Biso	1.000000	B
B32	1.0	0.451197	0.627990	0.024911	Biso	1.000000	B
B33	1.0	0.621554	0.752984	0.024911	Biso	1.000000	B
B34	1.0	0.509031	0.752984	0.024910	Biso	1.000000	B
B35	1.0	0.396519	0.752985	0.024911	Biso	1.000000	B
B36	1.0	0.566888	0.877990	0.024911	Biso	1.000000	B
B37	1.0	0.451197	0.877990	0.024911	Biso	1.000000	B
B38	1.0	0.621554	0.002984	0.024911	Biso	1.000000	B
B39	1.0	0.509031	0.002984	0.024910	Biso	1.000000	B
B40	1.0	0.396519	0.002985	0.024911	Biso	1.000000	B
B41	1.0	0.900222	0.127989	0.024911	Biso	1.000000	B
B42	1.0	0.784530	0.127990	0.024911	Biso	1.000000	B
B43	1.0	0.954887	0.252984	0.024911	Biso	1.000000	B
B44	1.0	0.842364	0.252984	0.024910	Biso	1.000000	B
B45	1.0	0.729852	0.252985	0.024911	Biso	1.000000	B
B46	1.0	0.900222	0.377990	0.024911	Biso	1.000000	B
B47	1.0	0.784530	0.377990	0.024911	Biso	1.000000	B
B48	1.0	0.954887	0.502985	0.024911	Biso	1.000000	B
B49	1.0	0.842364	0.502984	0.024910	Biso	1.000000	B
B50	1.0	0.729852	0.502985	0.024911	Biso	1.000000	B
B51	1.0	0.900222	0.627990	0.024911	Biso	1.000000	B
B52	1.0	0.784530	0.627990	0.024911	Biso	1.000000	B
B53	1.0	0.954887	0.752985	0.024911	Biso	1.000000	B
B54	1.0	0.842364	0.752984	0.024910	Biso	1.000000	B
B55	1.0	0.729852	0.752985	0.024911	Biso	1.000000	B
B56	1.0	0.900222	0.877990	0.024911	Biso	1.000000	B
B57	1.0	0.784530	0.877990	0.024911	Biso	1.000000	B
B58	1.0	0.954888	0.002984	0.024911	Biso	1.000000	B
B59	1.0	0.842364	0.002984	0.024910	Biso	1.000000	B
B60	1.0	0.729852	0.002985	0.024911	Biso	1.000000	B

##

For the relaxed GN sheet:

```
#======================================================================
# CRYSTAL DATA
#----------------------------------------------------------------------
data_VESTA_phase_1

_chemical_name_common                  'XCrySDen XSF file'
_cell_length_a                         12.807989
_cell_length_b                         12.330251
_cell_length_c                         19.994244
_cell_angle_alpha                      89.999504
_cell_angle_beta                       90.000038
_cell_angle_gamma                      90.000000
_cell_volume                           3157.605266
_space_group_name_H-M_alt              'P 1'
_space_group_IT_number                 1

loop_
_space_group_symop_operation_xyz
   'x, y, z'

loop_
   _atom_site_label
   _atom_site_occupancy
   _atom_site_fract_x
   _atom_site_fract_y
   _atom_site_fract_z
   _atom_site_adp_type
   _atom_site_B_iso_or_equiv
   _atom_site_type_symbol
   C1         1.0     0.887603     0.009720     0.028129    Biso  1.000000 C
   C2         1.0     0.776524     0.009722     0.028171    Biso  1.000000 C
   C3         1.0     0.720939     0.509711     0.027553    Biso  1.000000 C
   C4         1.0     0.609844     0.509708     0.027585    Biso  1.000000 C
   C5         1.0     0.554264     0.009719     0.028230    Biso  1.000000 C
   C6         1.0     0.443168     0.009719     0.028230    Biso  1.000000 C
   C7         1.0     0.387588     0.509708     0.027586    Biso  1.000000 C
   C8         1.0     0.443159     0.609720     0.027470    Biso  1.000000 C
   C9         1.0     0.554273     0.609720     0.027470    Biso  1.000000 C
   C10        1.0     0.609858     0.709739     0.027707    Biso  1.000000 C
   C11        1.0     0.720931     0.709730     0.027669    Biso  1.000000 C
   C12        1.0     0.776528     0.609727     0.027401    Biso  1.000000 C
   C13        1.0     0.887603     0.609722     0.027362    Biso  1.000000 C
   C14        1.0     0.943173     0.509714     0.027476    Biso  1.000000 C
   C15        1.0     0.943181     0.709738     0.027601    Biso  1.000000 C
   C16        1.0     0.887612     0.809737     0.028030    Biso  1.000000 C
   C17        1.0     0.943182     0.909723     0.028262    Biso  1.000000 C
   C18        1.0     0.776519     0.809740     0.028065    Biso  1.000000 C
```

C19	1.0	0.609856	0.909720	0.028365	Biso	1.000000	C
C20	1.0	0.720937	0.909719	0.028333	Biso	1.000000	C
C21	1.0	0.554269	0.809734	0.028133	Biso	1.000000	C
C22	1.0	0.443163	0.809734	0.028134	Biso	1.000000	C
C23	1.0	0.276500	0.709730	0.027666	Biso	1.000000	C
C24	1.0	0.387574	0.709739	0.027707	Biso	1.000000	C
C25	1.0	0.276492	0.509712	0.027550	Biso	1.000000	C
C26	1.0	0.220907	0.009723	0.028165	Biso	1.000000	C
C27	1.0	0.109828	0.009720	0.028122	Biso	1.000000	C
C28	1.0	0.054258	0.509714	0.027471	Biso	1.000000	C
C29	1.0	0.109828	0.609723	0.027355	Biso	1.000000	C
C30	1.0	0.054251	0.709738	0.027596	Biso	1.000000	C
C31	1.0	0.109820	0.809737	0.028023	Biso	1.000000	C
C32	1.0	0.054249	0.909723	0.028257	Biso	1.000000	C
C33	1.0	0.220913	0.809740	0.028059	Biso	1.000000	C
C34	1.0	0.276495	0.909719	0.028329	Biso	1.000000	C
C35	1.0	0.387576	0.909721	0.028365	Biso	1.000000	C
C36	1.0	0.220904	0.609727	0.027395	Biso	1.000000	C
C37	1.0	0.887609	0.409715	0.027713	Biso	1.000000	C
C38	1.0	0.776522	0.409712	0.027757	Biso	1.000000	C
C39	1.0	0.720931	0.109710	0.027967	Biso	1.000000	C
C40	1.0	0.609856	0.109715	0.027995	Biso	1.000000	C
C41	1.0	0.554277	0.409720	0.027814	Biso	1.000000	C
C42	1.0	0.443155	0.409720	0.027814	Biso	1.000000	C
C43	1.0	0.387576	0.109715	0.027995	Biso	1.000000	C
C44	1.0	0.943188	0.109713	0.027883	Biso	1.000000	C
C45	1.0	0.276500	0.109710	0.027963	Biso	1.000000	C
C46	1.0	0.220909	0.409712	0.027751	Biso	1.000000	C
C47	1.0	0.109822	0.409715	0.027706	Biso	1.000000	C
C48	1.0	0.054244	0.109713	0.027879	Biso	1.000000	C
C49	1.0	0.720942	0.309697	0.027924	Biso	1.000000	C
C50	1.0	0.609847	0.309692	0.027951	Biso	1.000000	C
C51	1.0	0.387585	0.309692	0.027951	Biso	1.000000	C
C52	1.0	0.943173	0.309698	0.027840	Biso	1.000000	C
C53	1.0	0.276490	0.309697	0.027920	Biso	1.000000	C
C54	1.0	0.054259	0.309698	0.027836	Biso	1.000000	C
C55	1.0	0.887595	0.209700	0.027778	Biso	1.000000	C
C56	1.0	0.776533	0.209700	0.027824	Biso	1.000000	C
C57	1.0	0.554263	0.209695	0.027880	Biso	1.000000	C
C58	1.0	0.443168	0.209696	0.027880	Biso	1.000000	C
C59	1.0	0.220898	0.209700	0.027819	Biso	1.000000	C
C60	1.0	0.109836	0.209700	0.027771	Biso	1.000000	C
